# In-Hospital Intravenous Thrombolysis Offers No Benefit in Mechanical Thrombectomy in Optimized Tertiary Stroke Center Setting

**DOI:** 10.1007/s00270-020-02727-8

**Published:** 2020-12-22

**Authors:** Juha-Pekka Pienimäki, Jyrki Ollikainen, Niko Sillanpää, Sara Protto

**Affiliations:** 1 Vascular and Interventional Radiology Center, Tampere University Hospital, Tampere, Finland; 2 Department of Neurology, Tampere University Hospital, Tampere, Finland; 3 Medical Imaging Center, Tampere University Hospital, PL2000, 33521 Tampere, Finland

**Keywords:** Ischemic stroke, Intravenous thrombolysis, Mechanical thrombectomy

## Abstract

**Purpose:**

Mechanical thrombectomy (MT) is the first-line treatment in acute stroke patients presenting with large vessel occlusion (LVO). The efficacy of intravenous thrombolysis (IVT) prior to MT is being contested. The objective of this study was to evaluate the efficacy of MT without IVT in patients with no contraindications to IVT presenting directly to a tertiary stroke center with acute anterior circulation LVO.

**Materials and Methods:**

We collected the data of 106 acute stroke patients who underwent MT in a single high-volume stroke center. Patients with anterior circulation LVO eligible for IVT and directly admitted to our institution who subsequently underwent MT were included. We recorded baseline clinical, laboratory, procedural, and imaging variables and technical, imaging, and clinical outcomes. The effect of intravenous thrombolysis on 3-month clinical outcome (mRS) was analyzed with univariate tests and binary and ordinal logistic regression analysis.

**Results:**

Fifty-eight out of the 106 patients received IVT + MT. These patients had 2.6-fold higher odds of poorer clinical outcome in mRS shift analysis (*p* = 0.01) compared to MT-only patients who had excellent 3-month clinical outcome (mRS 0–1) three times more often (*p* = 0.009). There were no significant differences between the groups in process times, mTICI, or number of hemorrhagic complications. A trend of less distal embolization and higher number of device passes was observed among the MT-only patients.

**Conclusions:**

MT without prior IVT was associated with an improved overall three-month clinical outcome in acute anterior circulation LVO patients.

**Supplementary Information:**

The online version of this article (10.1007/s00270-020-02727-8) contains supplementary material, which is available to authorized users.

## Introduction

Intravenous thrombolysis (IVT) has been proven to have a positive effect on patient survival and functional outcome in ischemic stroke if administered within a strict time window from symptom onset [1]. However, the effect of IVT is very limited on large vessel occlusions (LVO) thrombi [2]. Mechanical thrombectomy (MT) performed with modern methods is vastly superior in treating this patient group [3]. IVT prior to MT appears to have a favorable effect on clinical outcome [4–7]. However, some studies have reported no significant effect on the outcome [8–13]. Bellwald et al., moreover, found that there were more deaths at 3 months among the IVT-treated patients [14].

The objective of this study was to evaluate the efficacy of MT without IVT in patients with no contraindications to IVT presenting directly to a tertiary stroke center with acute anterior circulation LVO in comparison with patients who received both MT and IVT in the same setting.

## Methods

### Participants and Variables

We conducted an observational study on patients who underwent MT to treat anterior circulation acute LVO in a single high-volume stroke center. We collected the data of 479 consecutive patients having acute LVO (from ICA up to the M3 segment of the MCA) and no contraindications to IVT, who were treated with MT between October 2016 and October 2019, and had not been first evaluated in a referring hospital. All patients were admitted as MT candidates based principally on Finnish Prehospital Stroke Scale (FPSS) [15] scoring by the emergency services, or after clinical neurological examination in the emergency room or in case of in-hospital emergency in a ward. Patients underwent CT, and after treatment, decision was transferred immediately to an angiography suite for MT. Because of a highly standardized routine protocol with optimized in-hospital delays of less than an hour, neurologists on-call had the option to withhold from giving IVT, provided that the neurointerventional team was promptly available. Skipping IVT potentially further reduces the delay and the incidence of hemorrhagic complications, especially if a stent is implanted requiring initiation of antiplatelet medication. If the patient was given IVT, the bolus was administered on the CT table and the infusion was started on the way to the angiography suite and continued while MT was being performed. The infusion was stopped if satisfactory recanalization result (mTICI 2b-3) was obtained.

**Fig. 1:**
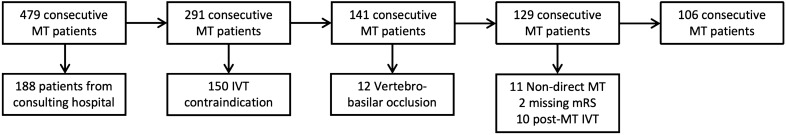
Flowchart showing the patient exclusion process

We identified ten cases where the effect of the thrombolytic agent was waited for before proceeding with MT. These patients were excluded because of the added delay to maintain comparability. Ten other patients received IVT after the MT to dissolve the remaining thrombi. They were excluded from the main analysis. Two patients could not be reached for the 3-month mRS control and were excluded. Finally after excluding patients who had contraindications to IVT, in total 106 patients met the inclusion criteria of whom 58 patients (55%) had IVT (flowchart in Fig. 1).

Baseline clinical characteristics included age, gender, and clinical risk factors for ischemic stroke (hypertension, diabetes, coronary heart disease, atrial fibrillation) collected from the patient records. National Institutes of Health Stroke Scale (NIHSS) score at the admission, process time points, mTICI (modified Thrombolysis in Cerebral Ischemia) grading evaluated with DSA at the end of the procedure, and procedural complications had been prospectively collected. A follow-up non-contrast-enhanced computed tomography (NCCT) was performed 24 h after MT. The clinical outcome measure was the modified Rankin Scale, evaluated 3 months after the stroke based on a follow-up visit or a phone interview by a neurologist. Shift analysis of mRS was the main outcome measure; the widely used dichotomization cutoffs and mortality were also analyzed. The CT imaging time point was recorded as the timestamp of the first scout image. The study was approved by the institutional review board and adhered to the Helsinki Declaration. A written consent was not deemed necessary by the review board.

### Imaging Parameters

Please see online supplementary material.

### Recanalization Therapies

All MT procedures were performed under conscious sedation. All procedures except one were performed with a balloon guide catheter. In 96% of cases, a stent retriever was used (Trevo®, Stryker, Salt Lake City, USA or EmbotrapII®, Neuravi, Galway, Ireland). An intermediate catheter was used for distal aspiration in 59% of cases in conjunction with stent retriever thrombectomy. IVT bolus of recombinant tissue plasminogen activator (rt-PA, Actilyse® 0.9 mg/kg, Boehringer-Ingelheim, Ingelheim, Germany) was administered after NCCT followed by infusion according to international guidelines.

### Statistics

The data were analyzed with SPSS version 25 (SPSS Inc., Chicago, IL). The analyses were performed using the maximum number of patients available with regard to missing data. Group comparisons were performed by using the Student *t* test, the Chi-squared test, the Fisher exact test, the Kruskal–Wallis test, and the Mann–Whitney *U* test according to the type and distribution properties of the variable studied. An mTICI score 2b-3 was considered a good recanalization result. Logistic regression analyses using dichotomized mRS as dependent variable were performed, and odds ratio (OR) with 95% confidence interval (CI) were calculated for each covariate. We performed shift analysis for mRS using ordinal regression analysis and calculated common odds ratio. A *p* value < 0.05 was considered statistically significant.

## Results

### Baseline and Technical Characteristics

Fifty-eight patients (55%) received IVT and MT, and 48 patients had MT only. Twenty-five percent of the patients had occlusion of terminus of internal carotid artery (ICA-T) and 57% in the first segment of median cerebral artery (M1). Isolated ICA occlusion with open anterior communicating artery was found in 5%, and the rest had M2 (14%) or M3 (2%) occlusions (occlusion site distribution in Supplementary Table 1).

**Table 1:** Demographic, baseline, and admission imaging characteristics of all patients by the recanalization therapy

Characteristic	All patients *n* = 106	IVT + MT *n* = 58	MT-only *n* = 48	*P*_1_
Age (y), mean (SD)	71 (12)	69 (12)	72 (11)	0.168
Female sex (%)	39 (37)	21 (36)	18 (38)	0.891
NIHSS, median (IQR)	15 (9)	16.5 (8)	14 (9)	0.580
ASPECTS, median (IQR)	10 (2)	9.5 (2)	10 (2)	0.995
Onset reperfusion (min), mean (SD)	158 (79)	150 (71)	166 (86)	0.458
CT groin (min), mean (SD)	26 (11)	26 (12)	25 (9)	0.739
Collateral score > 1, *n* (%)	79 (65)	41 (71)	28 (58)	0.184
Hypertension, *n* (%)	60 (57)	29 (50)	31 (65)	0.132
Diabetes, *n* (%)	24 (23)	13 (22)	11 (23)	0.951
Atrial fibrillation, *n* (%)	56 (53)	37 (64)	19 (40)	**0.013**
Coronary artery disease, *n* (%)	19 (18)	12 (21)	7 (15)	0.415

ASPECTS: Alberta Stroke Program Early CT Score, CT-groin: delay from first CT image to groin puncture in MT, IVT: intravenous thrombolysis, MT: mechanical thrombectomy, NIHSS: National Institutes of Health Stroke Scale, Onset-reperfusion: delay from onset of symptoms and recanalization in MT, P_1_: *p*-value between groups. Boldface denotes statistical significance

The median NIHSS at admission was 15 (IQR 9), and the mean delay from symptom onset to reperfusion (onset-to-reperfusion time) was 158 min (SD 79 min). The mean delay from CT imaging to groin puncture (CT-to-groin time) was 26 min (SD 11 min). The main baseline and admission imaging characteristics are summarized in Table 1.

The only significant difference in baseline characteristics (Table 1) between the IVT + MT and MT-only groups was found in the proportion of atrial fibrillation (64% vs. 40%, respectively, *p* = 0.013), which is very likely a coincidental finding, considering there is no logical explanation for this. Onset-to-reperfusion and CT-to-groin times were not significantly different (150 min vs. 166 min, *p* = 0.458, and 26 min vs. 25 min, *p* = 0.739, respectively). Thus, administration of IVT did not significantly prolong the CT-to-groin time. Almost the same proportion of patients achieved good recanalization (mTICI 2b-3) in both groups (91% vs. 92%, *p* = 0.958). In the MT-only group, there was a trend of fewer distal emboli (10% vs. 24%, *p* = 0.067), but more device passes were needed for a satisfactory recanalization result (mean 2.3 vs. 1.8 passes, *p* = 0.093). The IVT + MT group had a nonsignificantly lower frequency of hemorrhagic complications (21% vs. 29%, *p* = 0.313). Characteristics of the MT procedure and clinical outcome are summarized in Table 2.

**Table 2:** Characteristics of MT operation and clinical outcome between IVT + MT and MT-only groups

Characteristic	All patients *n* = 106	IVT + MT *n* = 58	MT-only *n* = 48	*P*_1_
mTICI 2b-3, *n* (%)	97 (92)	53 (91)	44 (92)	0.958
Distal embolus, *n* (%)	19 (18)	14 (24)	5 (10)	0.067
Number of passes, mean (SD)	2.1 (1.7)	1.8 (1.6)	2.3 (1.9)	0.093
ICH n (%)	26 (25)	12 (21)	14 (29)	0.313
ASPECT 24 h, median (IQR)	9 (3)	9 (3)	9 (4)	0.780
3-month mRS 0–1, *n* (%)	51 (48)	22 (38)	29 (60)	**0.021**
3-month mRS 0–2, *n* (%)	72 (68)	39 (67)	33 (69)	**0.868**
3-month mRS 6, *n* (%)	12 (11)	9 (16)	3 (6)	0.134

ASPECTS 24 h: Alberta Stroke Program Early CT Score 24 h after MT, Distal embolus: embolus further vessel territory, ICH: any intracranial hemorrhage in CT 24 h after MT, IVT: intravenous thrombolysis, mRS: modified Ranking Scale, MT: mechanical thrombectomy, mTICI: modified Thrombolysis In Cerebral Infarction score, mRS 0–1: excellent clinical outcome, mRS 0–2: good outcome, mRS 6: death, number of passes: number of passes of thrombectomy device, P_1_: *p* value between groups. Boldface denotes statistical significance

### The Distribution of 3-Month mRS and Factors Predicting the 3-Month Clinical Outcome

**Fig. 2:**
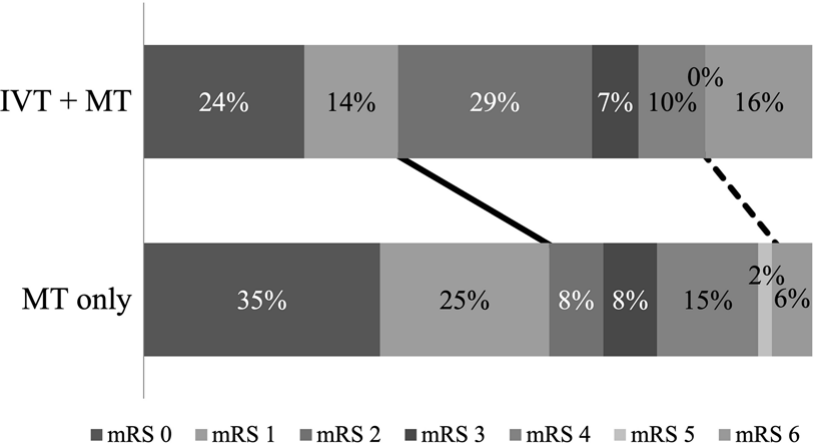
The distributions of 3-month mRS in the IVT + MT and MT-only groups. The line indicates the division between excellent clinical outcome (mRS 0–1, left-hand side) or not (right-hand side). The dotted line indicates the division between alive and dead (mRS 6, right-hand side). mRS: modified Rankin Scale

Figure 2 depicts shift analysis of the 3-month mRS between the two groups (*p* = 0.06 for overall difference). Analyzing the commonly used dichotomization cutoffs, the proportion of patients with good 3-month clinical outcome (mRS 0–2) was equivalent in the IVT + MT and MT-only groups (67% vs. 68%, *p* = 0.868), while the proportion of patients with excellent clinical outcome (mRS 0-) was significantly larger in the MT-only group (60% vs. 38%, *p* = 0.021), whereas the IVT + MT patients had a trend toward higher mortality (mRS = 6, 16% vs. 6%, *p* = 0.134).

The main established predictive factors of the 3-month clinical outcome were included as covariates in multivariable analyses along with variables having significant differences between the groups in univariable analyses.

Ordinal logistic regression analysis using 3-month clinical outcome as the dependent variable is detailed in Table 3. The common OR (shift analysis) between the IVT + MT and MT-only groups was 2.6 (95% CI 1.22–5.58, *p* = 0.01) with the IVT + MT patients having poorer clinical outcome. As expected from literature, each additional ten-minute delay in onset-to-reperfusion time increased the odds of poorer clinical outcome by 9% (OR = 1.09, 95% CI = 1.04–1.15, *p* < 0.001) and every additional NIHSS point by 8% (OR = 1.08, 95% CI = 1.02–1.15, *p* = 0.02).

**Table 3:** Ordinal logistic regression analysis of 3-month clinical outcome (mRS)

	O.R	Sig	95% C.I	
			Lower bound	Upper bound
Age	1.03	0.118	0.99	1.06
Onset-reper/10	1.09	** < 0.001**	1.04	1.15
NIHSS	1.08	**0.02**	1.02	1.15
AF	0.81	0.585	0.39	1.70
IVT + MT	2.61	**0.013**	1.22	5.58
mTICI 2b-3	0.69	0.540	0.20	2.30

Age: 1-year increase in age, AF: atrial fibrillation, C.I.: confidence interval, MT-only: mechanical thrombectomy patients without intravenous thrombolysis, mTICI: modified Thrombolysis In Cerebral Infarction score, mRS: modified Ranking Scale score, NIHSS: 1-point increase in National Institutes of Health Stroke Scale, Onset-reper/10: Delay from symptoms onset to achieve recanalization in MT (for every 10 min), O.R.: common odds ratio for worse outcome. Boldface denotes statistical significance. *Chi-square model significance* < *0.001*

Binary logistic regression analysis, using excellent 3-month clinical outcome (mRS 0–1) as dependent variable, is described in Supplementary Table 2. MT-only patients were over 3 times more likely to have excellent clinical outcome (OR = 3.37, 95% CI = 1.36 to 8.33, *p* = 0.009). The result of the analysis remained essentially unchanged after adding the site of occlusion, number of device passes, or detected distal embolization as an additional covariate. Further, the odds for excellent clinical outcome remained very similar when the ten patients who received IVT after MT were included to either the IVT + MT or the MT-only group (OR = 3.45, 95% CI = 1.44 to 8.23, *p* = 0.005, and OR = 2.65, 95% CI = 1.15 to 6.12, *p* = 0.022, respectively).

Using mRS = 6 (death) as the dependent variable (Supplementary Table 3), IVT increased the odds of death ninefold (OR = 8.98, 95% CI = 1.23 to 65.3, *p* = 0.030).

## Discussion

Intravenous thrombolysis with rt-PA and modern mechanical thrombectomy are well-proven treatments of acute ischemic stroke [1, 3]. The efficacy of IVT is limited in the treatment of large vessel occlusions [2]. The time from symptom onset to reperfusion is a critical determinant of clinical outcome [16]. Thus, the minimization of delays has a central role in the strategies to improve the outcome. Drip-and-ship protocols with IVT have been shown to improve the odds of good clinical outcome when direct access to MT is not available [17]. Protocols combining IVT to MT in a within-institution setting are less well studied [8–14]. Current guidelines state that IVT should always been given prior to MT if the patient is within the treatment time window and there are no contraindications [18–20].

In our study, treating the patient with both MT and IVT in comparison with proceeding directly to MT increased the odds of poorer 3-month clinical outcome 2.6-fold among patients eligible to receive IVT in a within-institution, minimal in-hospital delay, tertiary stroke center setting. To our knowledge, there are no previous studies reporting a signal that IVT might have this kind of limited unfavorable effect in conjunction with MT.

Some previous multicenter studies [4–6] have found that patients who received combined IVT and MT to treat LVO had better clinical outcome. In all these studies, IVT was always administered if not contraindicated. The average in-hospital delays were considerably longer than in our study. Further, the setup of these studies was different from ours, considering that they compared MT with IVT bridging therapy to MT in patients having contraindications for IVT. In our study, neither group had contraindications for IVT, and both groups had direct access to MT. On the other hand, in several other studies [8, 9, 11–14], no significant benefit in outcome was found when combining IVT and MT. Again, the average in-hospital delays in these studies were long. In three of the studies [8, 9, 11], IVT was always given if not contraindicated. In the studies by Weber et al. and Wang et al. [8, 12], the process times in the IVT + MT and MT-only groups were markedly different. Both Broeg-Morvay et al. and Bellwald et al. found higher mortality in matched-pair analyses when IVT was combined with MT, and IVT was not associated with better outcome [13, 14]. These two findings are in congruence with our results.

We found no significant differences in the frequency of symptomatic or non-symptomatic post-treatment ICH in the 24-h follow-up CT scans between the two groups, meaning that IVT appears to be safe and did not lead to more bleeding complications. However, both Broeg-Morvay et al. and Bellwald et al. found higher rates of hemorrhagic complications in the bridging therapy groups. We stopped the IVT infusion when satisfactory revascularization result was achieved in MT. Thus, a number of patients did not receive the full IVT dose, which may in part explain the difference. Moreover, we found no significant differences in process times or stroke severity at the admission, signaling that the administration of IVT does not add significant delay in an optimized tertiary hospital setting. We observed that the IVT + MT patients were more likely to have macroscopic distal emboli in comparison with the MT-only patients (24% vs. 10%, *p* = 0.067) similar to the findings of Yi et al., which potentially worsens the outcome [21]. On the other hand, fewer stent retriever passes were needed in the IVT + MT patients to achieve a satisfactory recanalization result (mean 1.8 vs. 2.3 passes, *p* = 0.093), which is in line with a report by Mistry et al. [4]. However, neither macroscopic distal embolization nor the number of retriever passes had a statistically significant effect on the overall technical or 3-month clinical outcome in multivariable analyses.

One potential unfavorable effect of IVT on the clinical outcome may be destabilization of the thrombus leading to fragmentation and microscopic distal embolization [21]. Alteplase can make the thrombus more fragile and prone to fragmentation during the procedure and thus increase the incidence of microinfarctions and post-ischemic microhemorrhages in the brain parenchyma. Microembolization and the sequelae are not readily detected in standard CT or DSA imaging. Patients with cerebral microhemorrhages in a follow-up MRI study after intravenous thrombolysis are known to have poorer clinical outcome [22–25]. A possible other mechanism not readily visible in follow-up CT, alteplase has been reported to have undesirable effects on the brain parenchyma due to neurotoxicity [26].

Two recent randomized trials (DIRECT-MT and SKIP trial) demonstrated non-inferiority but no advantage of MT only in comparison with IVT + MT in thrombectomy-capable centers. These trials had longer process times than in our study, which may diminish the efficacy of the MT-only approach [27, 28].

The inherent limitations of this study are the relatively small sample size that does not permit subgroup analyses of, for example, different occlusion sites and the observational and retrospective design that is prone to selection bias. The single, highly standardized tertiary stroke center with high average recanalization rate and minimal delay setup limits the generalizability of the findings. Further, our findings do not apply to drip-and-ship patients.

## Conclusions

MT without prior IVT was associated with improved overall three-month clinical outcome or was at least non-inferior in the treatment of acute anterior circulation LVO patients admitted directly to a tertiary stroke center. The ongoing randomized clinical trials, MRCLEAN_NoIV, SWIFT-Direct and DIRECT SAFE will help verifying or refuting this finding.

## Supplementary Information

Below is the link to the electronic supplementary material.

Supplementary file1 (DOCX 16 kb)

## Abbreviations

IVT: Intravenous thrombolysis
LVO: Large vessel occlusion
MT: Mechanical thrombectomy
mRS: Modified ranking scale
