# Supplementary material for: In-Hospital Intravenous Thrombolysis Offers No Benefit in Mechanical Thrombectomy in Optimized Tertiary Stroke Center Setting
Source: Cardiovasc Intervent Radiol. 2020 Dec 22;44(4):580–6. doi: 10.1007/s00270-020-02727-8 (PMC7987593; doi:10.1007/s00270-020-02727-8)
Supplement: Supplementary file 1 — Supplementary file1 (DOCX 16 kb) [file 270_2020_2727_MOESM1_ESM.docx]

**Supplementary material**

**Imaging parameters**

CT scans were obtained using a 64-row multidetector CT scanner (General Electric LightSpeed VCT, GE Healthcare, Milwaukee, WI, USA). Brain NCCT was performed using the parameters 120 kV with AUTO mA and SMART mA technique, noise index 3.3, collimation 4x5 mm, 40% adaptive statistical iterative reconstruction (ASIR), and rotation 0.5 s. Images were obtained axially (0.625mm thick slices) and then contiguous axial slices were reconstructed to the thickness of 5mm and coronal slices to the thickness of 2 mm. CTA was performed with helical technique using a scanning range from the aortic arch to the vertex of the skull. The imaging parameters were 100 kV, AUTO mA and SMART mA, noise index 9, 40% ASIR, collimation 40x0.625 mm, rotation 0.5 s, pitch factor 0.984. The contrast agent (iomeprol, 350 mg I/ml, IOMERON, Bracco, Milan, Italy) was administered via an antecubital vein with 18-gauge cannula using a double-piston power injector with a flow rate of 5ml/s using 70 ml contrast agent followed by a 50 ml saline flush. Automatic bolus triggering from the aortic arch was used. CTP was performed using the parameters 80 kV, 250 mA, 50% ASIR, collimation 8x5 mm, and rotation 0.4s. 272 slices covering a range of 80 mm were generated in 46 s using alternating toggle table protocol to increase the z-axis coverage. Contiguous slices were reconstructed to a thickness of 5 mm at even intervals. The contrast agent (IOMERON 350 mg I/ml) was administered via an antecubital vein with an 18-G cannula using a double-piston power injector with flow rate of 5ml/s using 40 ml of contrast agent followed by a 40 ml saline flush. Digital subtraction angiographic images were obtained using the Artis Z angiographer (Siemens, Munich, Germany) using the parameters 102 kV, AUTOmA and SMARTmA**.**

**Supplementary Table 1: Division of occlusion sites within IVT+MT and MT only groups**

ICA: internal carotid artery (with open anterior communicating artery), ICA-T: terminus of internal carotid artery, IVT+MT: intravenous thrombolysis with mechanical thrombectomy: MT only: mechanical thrombectomy without intravenous thrombolysis, M1: first segment of middle cerebral artery, M2: second segment of middle cerebral artery: M3: third segment of middle cerebral artery, P_1_: *p*-value between groups

| Occlusion site | All patients  n=106 | IVT + MT  n=58 | Only MT  n=48 | P_1_ |
| --- | --- | --- | --- | --- |
| ICA, n (%) | 5 (5) | 3 (5) | 2 (4) | 0.808 |
| ICA-T | 26 (25) | 11 (19) | 15 (31) | 0.143 |
| M1 | 58 (57) | 33 (57) | 25 (52) | 0.620 |
| M2 | 15 (14) | 10 (17) | 5 (10) | 0.316 |
| M3 | 2 (2) | 1 (2) | 1 (2) | 0.892 |

**Supplementary Table 2: Binary logistic regression analysis of excellent 3-month clinical outcome (mRS 0-1)**

AF: atrial fibrillation, C.I.: confidence interval, MT-only: mechanical thrombectomy patients without intravenous thrombolysis, mTICI: modified Thrombolysis In Cerebral Infarction score, mRS: modified Ranking Scale score, NIHSS: National Institutes of Health Stroke Scale, Onset-reper/10: Delay from symptoms onset to acheved recanalization in MT (for every 10 minutes), O.R.:odds ratio for excellent clinical outcome (mRS 0-1). Boldface denotes statistical significance. *Chi-square model significance 0.007, Hosmer and Lemeshow test 0.893, Cox and Snell significance 0.155*

|  |  |  | 95% C.I. | |
| --- | --- | --- | --- | --- |
|  | O.R. | Sig. | Lower Bound | Upper Bound |
| Age | 0.99 | 0.637 | 0.95 | 1.03 |
| Onset-reper/10 | 0.93 | **0.025** | 0.87 | 0.99 |
| NIHSS | 0.92 | **0.037** | 0.85 | 1.00 |
| AF | 1.26 | 0.610 | 0.52 | 3.03 |
| MT-only | 3.37 | **0.009** | 1.36 | 8.33 |
| mTICI 2b-3 | 3.00 | 0.207 | 0.54 | 16.54 |

**Suplementary Table 3: Binary logistic regression analysis of death (mRS 6)**

AF: atrial fibrillation, C.I.: confidence interval, MT only: mechanical thrombectomy patients without intravenous thrombolysis, mTICI: modified Thrombolysis In Cerebral Infarction score, mRS: modified Ranking Scale score, NIHSS: National Institutes of Health Stroke Scale, Onset-reper/10: Delay from symptoms onset to acheved recanalization in MT (for every 10 minutes), O.R.:odds ratio for excellent clinical outcome (mRS 0-1), *Chi-square model significance <0.001, Hosmer and Lemeshow test 0.490, Cox and Snell significance 0.206*

|  |  |  | 95% C.I. | |
| --- | --- | --- | --- | --- |
|  | O.R. | Sig. | Lower Bound | Upper Bound |
| Age | 1.17 | **0.005** | 1.05 | 1.31 |
| Onset-reper/10 | 1.13 | **0.021** | 1.02 | 1.25 |
| NIHSS | 1.13 | 0.125 | 0.97 | 1.33 |
| AF | 1.81 | 0.482 | 0.11 | 2.88 |
| IVT+MT | 8.98 | **0.030** | 1.23 | 65.3 |
| mTICI 2b-3 | 1.32 | 0.855 | 0.06 | 28.0 |
